# Association Between the Serum Uric Acid to Albumin Ratio and the No-Reflow Phenomenon After Percutaneous Coronary Intervention (PCI): A Systematic Review and Meta-Analysis

**DOI:** 10.7759/cureus.81712

**Published:** 2025-04-04

**Authors:** Mustafa B Ozbay, Serhat Degirmen, Aysenur Gullu, Bede N Nriagu, Mohammed Omer, Yasin Özen, Cagri Yayla

**Affiliations:** 1 Internal Medicine, Penn Medicine Princeton Medical Center, Plainsboro, USA; 2 Internal Medicine, New York Medical College, Metropolitan Hospital Center, New York, USA; 3 Internal Medicine, North Alabama Medical Center, Florence, USA; 4 Internal Medicine, Hurley Medical Center, Flint, USA; 5 Cardiology, Faculty of Medicine, Selcuk University, Konya, TUR; 6 Cardiology, Ankara City Hospital, University of Health Sciences, Ankara, TUR

**Keywords:** albumin, meta-analysis, no-reflow, pci, uric acid

## Abstract

The no-reflow phenomenon (NR) following percutaneous coronary intervention (PCI) is an unpredictable complication linked to increased in-hospital mortality and adverse cardiovascular outcomes. Reliable predictors of NR remain unclear. This systematic review and meta-analysis aimed to evaluate the association between serum uric acid to albumin ratio (UAR) and NR development after PCI. A comprehensive search of MEDLINE, Cochrane, and EMBASE databases identified observational studies assessing this relationship. Studies that provided multivariate regression analyses to determine whether UAR is an independent predictor of NR were included, and pooled odds ratios (OR) were calculated using random effects models. Three studies with a total of 2,199 patients were included. The pooled analysis demonstrated a significant association between higher UAR and an increased risk of NR (OR: 3.04; 95% CI: 2.26-4.10; p < 0.00001; I² = 0%). These findings indicate that a higher serum UAR is independently associated with NR development and may serve as a useful, easily measurable predictor in clinical practice.

## Introduction and background

The no-reflow phenomenon (NR) following percutaneous coronary intervention (PCI) is a complex and often unpredictable complication that can significantly impact patient outcomes, including increased in-hospital mortality, malignant arrhythmias, and heart failure [1]. The incidence of NR varies widely, ranging from 3% to 22%, and its pathophysiology is multifactorial, involving mechanisms such as microvascular injury, distal embolization, and reperfusion myocardial injury [1,2]. Despite extensive research, the precise causes and reliable predictors of NR remain unclear [3].

Recent studies have suggested that serum biomarkers may offer insights into predicting NR risk [4-7]. Among these biomarkers, the serum uric acid (UA) to albumin ratio (UAR) has gained attention due to its association with systemic inflammation and oxidative stress, both of which play critical roles in cardiovascular diseases [8]. UA contributes to endothelial dysfunction, inflammation, and thrombus formation, while albumin exhibits anti-inflammatory and antioxidant properties [8,9]. The combined effect of these two biomarkers, as represented by UAR, has shown promise in reflecting the severity of systemic inflammation and oxidative stress in patients with coronary artery disease (CAD) [10,11].

This systematic review and meta-analysis aims to investigate the association between the serum UAR and the development of NR following PCI. We systematically evaluated observational studies to assess whether UAR may be used as an independent predictor of NR in patients undergoing PCI.

## Review

Methods

Search Strategy

This study was conducted following the guidelines outlined in the Preferred Reporting Items for Systematic Reviews and Meta-Analyses (PRISMA) 2020 checklist and the Meta-Analysis of Observational Studies in Epidemiology (MOOSE) statement [12,13]. The protocol was prospectively registered in the International Prospective Register of Systematic Reviews (PROSPERO) database under the registration ID “CRD42025644721”. A systematic search was conducted across the MEDLINE (via PubMed), Cochrane, and EMBASE databases. The references of eligible papers and systematic reviews were also examined to identify additional relevant studies. The complete search strategy for each database can be found in the Supplemental Material. The database search was performed up to February 10, 2025.

Eligibility Criteria

We limited inclusion in this meta-analysis to studies meeting the following eligibility criteria: (1) observational studies, either prospective or retrospective; (2) comparing patients who developed NR after PCI with those who did not; and (3) conducted multivariate regression analyses to determine if the uric acid to albumin ratio (UAR) is an independent predictor of NR. We excluded studies with overlapping patient populations, studies published only as conference abstracts, case or case series reports, and studies published in languages other than English.

Data Extraction and Study Outcome

Three investigators (MBO, SD, and AG) independently conducted the data search, selected the studies, and extracted relevant data from the included studies. Disagreements were resolved through author consensus after reviewing the full article and the eligibility criteria with the senior author (CY).

Quality Assessment

For observational studies, we used the risk of bias in non-randomized studies of interventions (ROBINS-I) tool [14]. The risk of bias was performed by two independent investigators (MBO and SD) for every study included. Disagreements were resolved with the senior author (CY).

Data Analysis

We conducted all data analysis following the recommendations outlined by Cochrane [14]. Binary endpoints were summarized using the Mantel-Haenszel test with a random effects model odds ratio (OR) and 95% confidence interval (CI) as a measure of effect size. We used the Der Simonian-Laird method to implement the random effects model. All studies analyzed the UAR as a continuous variable, and ORs were extracted to reflect the change in outcomes per one-unit increase in the serum UAR. We assessed for heterogeneity using Cochrane’s Q statistic and Higgins and Thompson's I^2^ statistic. P values inferior to 0.10 and I2 > 25% were considered significant for heterogeneity. We performed sensitivity analyses using the "leave-one-out" approach. Statistical analyses were performed using Review Manager 5.4 (The Nordic Cochrane Centre, The Cochrane Collaboration, Denmark) and R software (version 4.2.2; R Development Core Team, Vienna, Austria).

Results

**Figure 1:**
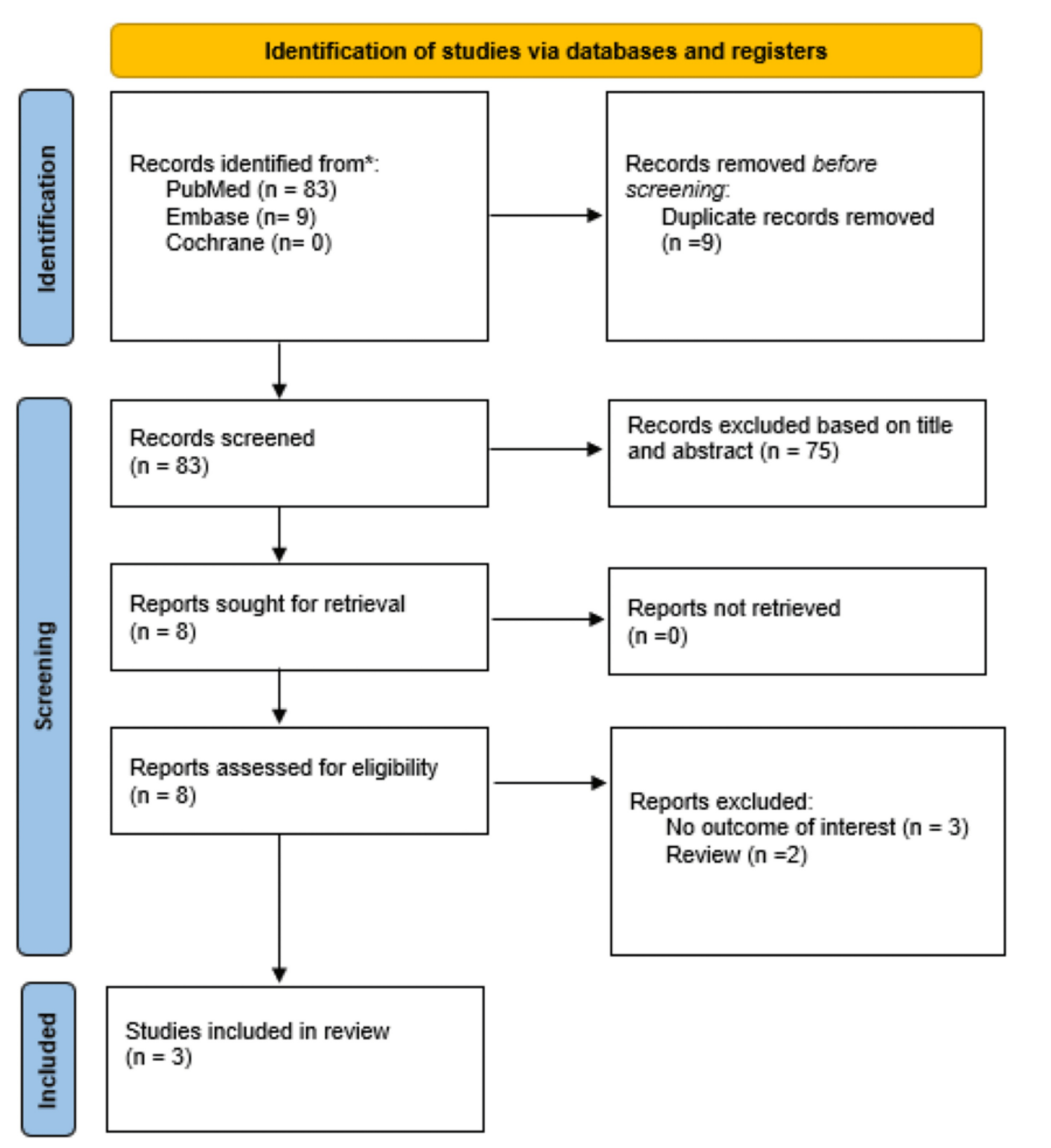
PRISMA flowchart of the search strategy PRISMA: Preferred Reporting Items for Systematic Reviews and Meta-Analyses

As detailed in Figure 1, our initial search yielded 92 records. After the removal of duplicate reports, we reviewed 83 studies in full text. Three studies met our inclusion criteria, encompassing 2,199 patients [4-6], of whom 294 (13.4%) were in the NR group and 1905 (86.6%) were in the no NR group. One study included patients presenting with acute ST-segment elevation myocardial infarction (STEMI) [4], and two studies included patients with non-ST-elevation myocardial infarction (NSTEMI) [5,6]. Further characteristics of the included studies are reported in Table 1.

**Table 1:** Baseline characteristics of the included studies ^a^mean or median. CAD: coronary artery disease; CKD: chronic kidney disease; DM: diabetes mellitus; HTN: hypertension; LVEF: left ventricular ejection fraction; NA: non-available; NR: no-reflow; NSTEMI: non-ST-elevation myocardial infarction; STEMI: ST-elevation myocardial infarction Sources: Refs. [4-6]

Study	Population	No. of patients, NR/no NR	Female, % NR/no NR	Age^a^, y NR/no NR	DM, % NR/no NR	HTN, % NR/no NR	Current smoker, % NR/no NR	History of CAD, % NR/no NR	LVEF^a^, NR/no NR	CKD, % NR/no NR
Çınar et al. 2023 [4]	Acute STEMI	91/747	28.7/24.6	61/62	28.6/20.1	50.5/48.1	64.8/59.6	23.1/18.9	45/45	NA
Kalyoncuoglu et al. 2025 [5]	NSTEMI	153/848	22.2/20.4	62.3/59.5	23.5/21.0	42.5/48.0	39.9/26.9	17.6/20.6	44.9/47.3	0
Nurkoc et al. 2024 [6]	NSTEMI	50/310	36/29	67.3/62.1	52/36.8	54/49	36/49	22.0/24.5	45/55	0

**Figure 2:**
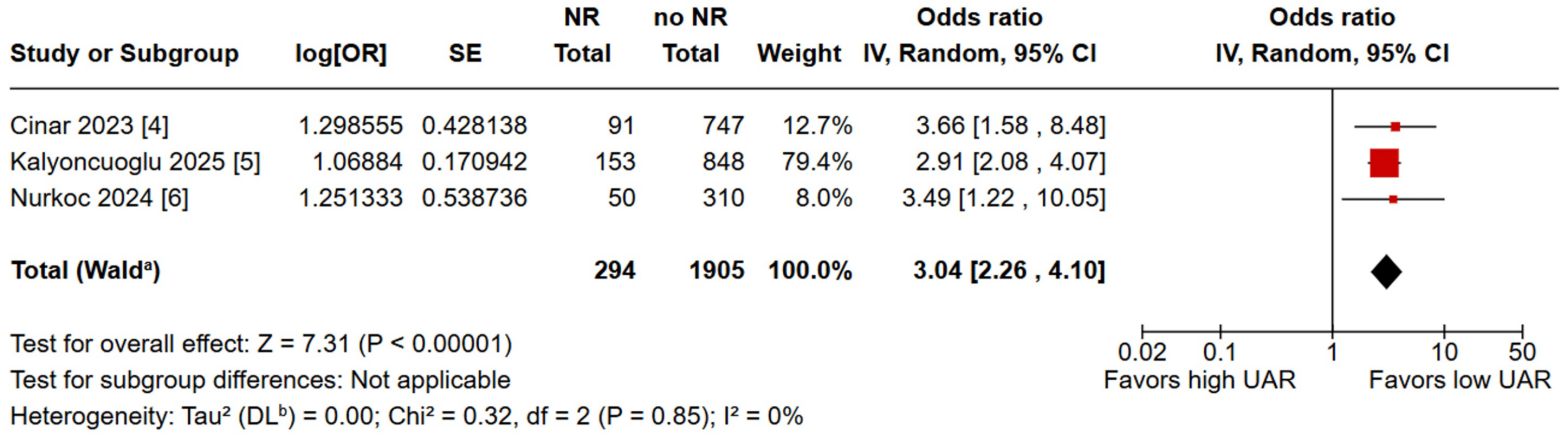
A higher uric acid to albumin ratio had a significantly higher rate association of no-reflow in patients undergoing percutaneous coronary intervention Sources: Refs. [4-6]

**Figure 3:**
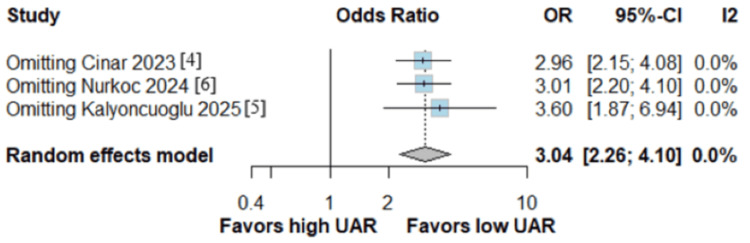
The uric acid to albumin ratio showed a consistent risk effect in the leave-one-out analysis Sources: Refs. [4-6]

A pooled analysis of 294 patients with NR and 1,905 with no NR showed a significantly higher rate association of NR with a higher UAR in patients undergoing PCI (OR: 3.04; 95% CI: 2.26 to 4.10; p < 0.00001; I^2^ = 0%; Figure 2). The overall risk of bias in observational studies was rated as moderate (Table 2). In the leave-one-out analysis, the UAR showed a consistent risk effect through all iterations (Figure 3).

**Table 2:** Risk of bias summary for non-randomized studies (ROBINS-I) Sources: Refs. [4-6]

Study	Bias due to confounding	Bias in selection of participants	Bias in classification of interventions	Bias due to deviations from intended interventions	Bias due to missing data	Bias in measurement of outcomes	Bias in selection of the reported result	Overall risk of bias judgement
Cinar et al. 2023 [4]	Moderate	Low	Low	Low	Moderate	Low	Moderate	Moderate
Kalyoncuoglu et al. 2025 [5]	Moderate	Moderate	Low	Low	Moderate	Moderate	Low	Moderate
Nurkoc et al. 2024 [6]	Moderate	Low	Low	Low	Moderate	Low	Moderate	Moderate

Discussion

In this systematic review and meta-analysis of three studies involving 2,199 patients [4-6], we compared patients who developed NR after PCI with those who did not. The main finding from the pooled analyses was that a higher UAR is associated with higher rates of NR development in patients who underwent PCI.

Previous studies exploring the UAR as a potential marker have consistently demonstrated its significant association with the NR phenomenon in patients undergoing PCI [4-6]. Çınar et al. [4] showed that a higher UAR was an independent predictor of NR in STEMI patients, outperforming its individual components. Nurkoç et al. [6] also found that, in NSTEMI patients, the UAR was an independent predictor of NR, showing a significant correlation with higher SYNTAX scores, neutrophil-to-lymphocyte ratios, and lower left ventricular ejection fractions. Similarly, Kalyoncuoglu et al. [5] reported a significant association between a higher UAR and NR in NSTEMI patients undergoing PCI.

NR following PCI has a significant association with higher in-hospital mortality, malignant arrhythmias, and heart failure [1]. The incidence of NR can vary between 3% and 22% [1,2]. NR is believed to be multifactorial, with its etiology remaining unclear [3]. The mechanisms considered to contribute to the pathophysiology of NR include distal embolization of plaque and thrombus, microvascular injury, reperfusion myocardial injury caused by oxygen radical production, myocardial necrosis, the release of active tissue factors from dissected plaque, vasoconstriction due to increased alpha-adrenergic tone, thromboxane A2 from platelets, and the release of serotonin [14].

Inflammation is known to play a role in the development of atherosclerosis [15]. Albumin serves multiple functions, including transporting various physiologically active substances, buffering pH, and, most notably, exhibiting anti-inflammatory and antioxidant properties [16]. Albumin is a negative acute-phase protein; therefore, its levels are associated with the severity of inflammation in critically ill patients [8]. Decreased serum albumin is associated with endothelial dysfunction, elevated blood viscosity, and platelet aggregation [9]. Lower albumin levels are associated with worse outcomes in patients with coronary artery disease [17-19].

UA can crystallize and trigger local inflammatory responses by forming monosodium urate crystals in various organs [20]. Inflammatory activities associated with UA crystals are significantly increased in patients with CAD [21]. Human atherosclerotic plaques contain substantial amounts of UA, and elevated serum levels can enhance thrombus formation through purine metabolism [22,23]. The UAR has been investigated as a potential new marker of inflammation and oxidative stress in patients with cardiovascular conditions [24,25]. The superiority of the UAR over individual uric acid and albumin levels in reflecting systemic inflammation has been demonstrated [10,11]. This has prompted the exploration of a potential association between the development of the UAR and NR [4-6].

To the best of our knowledge, this is the first meta-analysis to investigate the association between the NR and UAR. Our findings suggest that the UAR is an independent predictor of NR in patients undergoing PCI and offers a practical tool for estimating the risk of NR in these patients. Furthermore, the correlation between the NR and UAR suggests that preventive measures could be implemented to mitigate complications in this population [4-6].

Our study has limitations. First, only three studies were included in the meta-analysis, which precludes further sensitivity analyses such as funnel plot analysis and Egger’s test; the I² statistic indicated no significant heterogeneity (I² = 0%), although this could be due to the small number of studies. Second, all included studies were observational. These study designs are susceptible to confounding variables. However, multivariate regression analyses were used to adjust for potential confounders in order to minimize bias, although residual confounding may still influence the results. Larger, high-quality clinical trials are needed to confirm these findings and explore the mechanisms behind this association.

## Conclusions

This systematic review and meta-analysis provides evidence that a higher UAR is independently associated with the development of NR following PCI. Our findings suggest that the UAR may serve as a simple and practical biomarker for identifying patients at higher risk of NR, potentially aiding in risk stratification and early intervention. Given the role of inflammation and oxidative stress in NR pathophysiology, the association between UAR and NR highlights the need for further investigation into the underlying mechanisms and potential therapeutic strategies. However, due to the limited number of included studies and the observational nature of the data, larger prospective studies are required to validate these findings and establish the clinical utility of the UAR in predicting NR after PCI.

## Appendices

Full search strategy: ("No-Reflow Phenomenon"[Mesh] OR "no reflow phenomenon" OR "no-reflow" OR "microvascular obstruction" OR "NR") AND ("UAR" OR "Uric Acid/Albumin Ratio" OR "UA/albumin" OR "SUA to albumin ratio")
